# Improved Mannanase Production from *Penicillium occitanis* by Fed-Batch Fermentation Using Acacia Seeds

**DOI:** 10.5402/2011/938347

**Published:** 2011-10-24

**Authors:** Monia Blibech, Raoudha Ellouz Ghorbel, Fatma Chaari, Ilyes Dammak, Fatma Bhiri, Mohamed Neifar, Semia Ellouz Chaabouni

**Affiliations:** ^1^Unité Enzymes et Bioconversion, Ecole National d'ingénieurs de Sfax, Université de Sfax, Route de Soukra 3038, Sfax, Tunisia; ^2^Unité de Service Commun Bioréacteur Couplé à un Ultrafiltre, Ecole Nationale d'Ingénieurs de Sfax, Université de Sfax, Route de Soukra 3038, Sfax, Tunisia

## Abstract

By applying a fed-batch strategy, production of *Penicillium occitanis* mannanases could be almost doubled as compared to a batch cultivation on acacia seeds (76 versus 41 U/mL). Also, a 10-fold increase of enzyme activities was observed from shake flask fermentation to the fed-batch fermentation. These production levels were 3-fold higher than those obtained on coconut meal. The high mannanase production using acacia seeds powder as inducer substrate showed the suitability of this culture process for industrial-scale development.

## 1. Introduction

In recent years, mannanases (1,4-*β*-D-mannan mannohydrolase, EC 3.2.1.78) have gained increasing attention because of their various biotechnological applications in the food, feed, coffee extraction, oil drilling, detergent, as well as pulp and paper industries [1–4]. They can also be used in the production of mannooligosaccharides, which were reported to be excellent prebiotics stimulating growth of beneficial intestinal microorganisms [5, 6].

Many microorganisms have been reported as mannanase producers. Mannanases from fungi such as *Aspergillus sp*ecies [3, 7, 8], *Trichoderma reesei* [9], *Sclerotium rolfsii *[10], and *Penicillium occitanis* [11] have been well described. Fungal mannanases are generally produced in the presence of different mannan-rich substrates. These included locust bean gum (LBG) [7], guar gum [12], konjac flour [13], and copra meal [3, 14]. 

Fed-batch culture is commonly used in microbial fermentation. The main purpose of employing this fermentation method was to remove the repressive effects of a rapidly utilized carbon source, to reduce the effect of toxic medium constituents and to extend the process product formation stage as long as possible [15].

*Penicillium occitanis* was previously described as an outstanding producer of cellulolytic and hemicellulolytic enzymes [11, 16]. The production of cellulase by this strain under fed-batch culture was also studied [16]. However, there are very few reports on fed-batch fermentation for efficient mannanase production [17]. The aim of the present study was to develop a fed-batch process for enhanced production of mannanase by* P. occitanis* using acacia seeds as a novel inducer of substrate.

## 2. Materials and Methods

### 2.1. Strain

The *Penicillium occitanis* mutant Pol6 was supplied by Cayla Co. (Toulouse, France). The Pol6 strain is a hypercellulolytic mutant selected by Jain et al. [18] after eight rounds of mutagenesis from the CL100 wild-type strain.

### 2.2. Substrates

Locust bean gum (LBG) was procured from Sigma (St. Louis, Mo, USA). Commercial coconut meal was procured from local market in Tunisia. Samples of acacia were collected during April-May, in 2010, from Sfax (Tunisia). The dried seeds were transferred to a mortar, crushed with a pestle, and sieved with a standard 0.5 and 1 mm sieves to obtain two different particle sizes (P1 (<0.5 mm ) and P2 ( 0.5-1 mm)). The chemical composition of coconut and acacia seeds is presented in Table 1.

### 2.3. Inoculum Preparation

Mandels and Weber's medium modified by Ellouz Chaabouni et al. [16] was used for the production of *β*-mannan-degrading activity of *P. occitanis* Pol6. Its composition was (g/L) KH_2_PO_4_, 2; NaNO_3_, 5; MgSO_4_·7H_2_O, 0.3; CaCl_2_, 0.3; yeast extract, 1 g; tween 80, 1 mL/L; trace elements solution, 1 mL/L. The trace elements solution contained (in g/L) CoCl_2_, 2; MnSO_4_·H_2_O, 1.6; ZnSO_4_· H_2_O, 1.4; FeSO_4_· 7H_2_O, 5.0. The initial pH value was adjusted to 5.5 with orthophosphoric acid (2 M) before sterilisation at 121°C for 20 min. The medium (100/500 mL Erlenmeyer flasks), added with glucose at 2 g/L as a carbon substrate, was inoculated with a mycelial culture grown on potato dextrose agar plates for 1 week. The liquid culture was then incubated for 3 days at 30°C on a rotary shaker at 180 rpm. 

### 2.4. Fermentation Conditions

#### 2.4.1. Erlenmeyer Cultivation

Cultures with carbon mannan sources (coconut meal, acacia seeds at two different particle sizes) were carried out in Erlenmeyer flasks (50/250 mL). 1 mL sample of the above inoculum was added to each flask. Samples were removed after 6 days and analysed for mannanase activity.

#### 2.4.2. Batch and Fed-Batch Cultures

A 30-L fermenter (Infors S-000111297, Suisse) was used for all experiments. The fermenter was equipped with automatic control of agitation, temperatures, pH, dissolved oxygen concentration (pO2), and foaming. Batch cultures were carried out by adding a 7% inoculum to 7 L sterile medium in a presterilized fermenter (121°C, 30 min). The pH was maintained at 5.5 by adding sodium hydroxide (2 M) or orthophosphoric acid (2 M). The temperature was kept constant at 30°C. Antifoam (1%) was added when required. Aeration automatically varied from 0.1 to 1.0 volume of air per fluid volume per min (v/m) to maintain a PO_2_ of 20% of air saturation. 

The fed-batch culture was started with a batch growth phase, followed by a feeding phase. Acacia seeds powder were added by intermittent feeding at a final concentration of 20 g/L. The feeding procedure of the fermenter with coconut meal or acacia seeds powder (0.75 g/h) began after 3 days. Broth samples were taken regularly during the course of fermentation. After centrifugation (7000 xg, 10 min), supernatants were used to determine mannanase activities. 

### 2.5. Enzyme Assay

*β*-mannanase was determined as described by Blibech et al. [11]. The release of reducing sugars in 5 min at 100°C was measured as mannose equivalents using the dinitrosalicylic acid (DNS) method [19].

### 2.6. Estimation of Biomass Concentration

Cell growth was followed indirectly by determination of the protein content of the cultivated material by the Kjeldahl method [20]. 

## 3. Results and Discussion

### 3.1. Mannanase Production in Shake Flask Culture

Commercial mannans such as locust bean gum, guar gum, and konjac flour substrates were largely used for fungal mannanase production [7, 12, 13]. In the hope of improving the economics of the enzyme production for large scale, it is interestingly to use cheap substrates as coconut meal [15], date seeds [21], and carob pods [11]. 

Acacia seeds contained considerable amounts of carbohydrate (53.4%) and organic nitrogen sources (23.37%) (Table 1) essential for growth and protein biosynthesis. In this study, we tested acacia seeds as a novel substrate for mannanase production under submerged conditions, and we compared this production with coconut meal, extensively used as economical inducer of substrate [3, 14].

**Figure 1:**
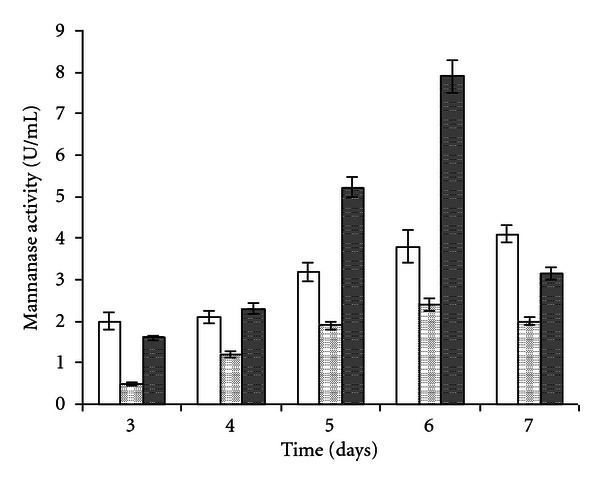
Mannanase production by *Penicillium occitanis* at Erlenmeyer scale using coconut meal (empty square) and acacia seeds powder with different particle size range: (light dots square) P1 (<0.5 mm) and (heavy dots square) P2 (0.5-1 mm) as substrates. Data presented is the average of three experiments and *error bars* represent the standard deviations.

The mannanase production data presented in Figure 1 revealed that both substrates (coconut meal and acacia seeds) support *P. occitanis* growth and enzyme production. Maximum mannanase yields were noticed at, 6th day of incubation (about 8 U/mL), and further increase in fermentation time resulted in the reduction of mannanase activities.

The enzyme production pattern, however, varied with acacia particle size. Enhanced mannanase-production (7.9 ± 0.15 U/mL) was recorded with substrate particles size ranging from 0.5 to 1 mm. It was three times higher than the production yield obtained with substrate particles of an average size <0.5 mm. This may be due to an increase in interparticle porosity which provided better aeration and efficient nutrient availability for mycelium growth.

The mannanase yield obtained on acacia seeds in shake flask culture was higher than those obtained by other mannanase-producer fungi cultivated in various agricultural substrates/byproducts-based media (Table 2).

### 3.2. Mannanase Production by Batch and Fed-Batch Processes

Batch fermentation experiment was carried out in 30 L fermentor scale. The mannanase activity and productivity reached the maximum of 41.1 U/mL (at 72 h) and 571.1 U/l/h, respectively, when using acacia seeds as substrate. These values were threefold higher than those obtained using coconut meal (Table 3). The significant increase of mannanase production by batch fermentation compared to the shake flask culture could be explained by the improved oxygen transfer in the bioreactor. Oxygen limitation could be a serious problem in the shaken flask cultivations due to the highly non-Newtonian medium that is caused by the filamentous growth of the fungus [17].

**Figure 2:**
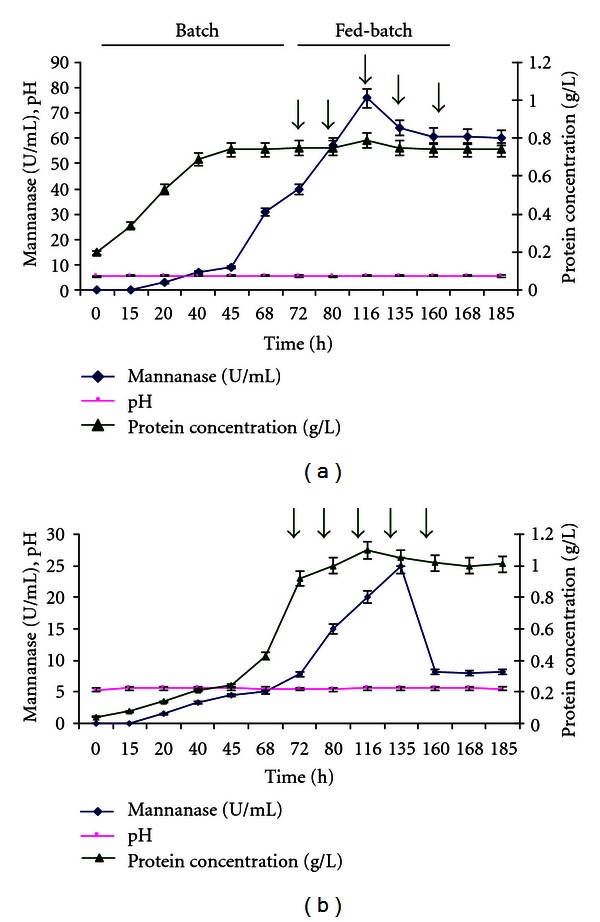
Fermentation fed with acacia seeds powder (a) and coconut meal (b). The initial carbon source was 20 g/L, and feeding was carried out from 72 to 185 h at a rate of 0.75 g l^−1^h^−1^.

In order to improve the production of mannanase on acacia seeds, fed-batch fermentation was performed at a feed rate of 0.75 g/l/h. The profile of mannanase activity, cell growth, and pH was shown in Figure 2(a). The mannanase production increased with feeding until approximately 120 h of growth and then stayed relatively constant for the remainder of the cultivation. In contrary to fed-batch culture using acacia seeds, the mannanase production on coconut meal decreased drastically in the latter phase of the cultivation (Figure 2(b)). This result may be explained by the accumulation of the copra oil content which had an inhibitory effect on fungal growth and mannanase production as previously reported [3, 14].

The highest mannanase activity and productivity obtained by fed-batch fermentation using acacia seeds as substrate were 76 ± 3.1 U/mL and 649.5 ± 53.6 U/l/h respectively. These values were about 10-fold higher than those obtained in the Erlenmeyer flask cultures. These results were in agreement with others; for example, Gro*β*windhager et al. [17] reported an increase in mannanase production by almost sixfold (462 U/mL) by applying a fed-batch strategy in which a glucose solution was continuously fed to a culture of *Sclerotium rolfsii*.

## 4. Conclusions

The results obtained in the present study indicate high mannanase production and productivity by culturing *P. occitanis* in fed-batch using acacia seed as substrate. The highest mannanase activity reached 76 ± 3.11 U/mL which was about 10-fold in the shake flask culture and about three-fold in coconut meal. So, acacia seeds could be used as a less expensive substrate for an efficient mannanase production at industrial scale.

## Figures and Tables

**Table 1:** Chemical composition of acacia seed powder (% dry matter) [22] and coconut meal [23].

	Acacia seeds	Coconut meal
Dry matter	94.48	52.29
Ash	3.97	1.15
Crude protein	23.37	7.10
Crude fat	6.66	62.64
Carbohydrate	53.4	29.1
Hemicellulose	7.20	6.37
Cellulose	6.9	7.09
Lignin	1	6.69

**Table 2:** Mannanase activities obtained by several fungi under submerged fermentation.

Organism	Carbon source	Activity (U/ml)	References
*Aspergillus niger gr*	Copra meal	1.966	[14]
*A. flavus gr*	Copra meal	1.325	[14]
*A. niger *	Palm kernel cake	2.48	[24]
*Sclerotium rolfsii*	Palm kernel cake	3.16	[24]
*Trichoderma harzianum* T4	Wheat bran	8.20	[25]
*Thermomyces lanuginosus*	Corn cobs (coarse)	0.3	[10]
*Schizophyllum commune*	Corn cobs (coarse)	3.24	[10]
*S. commune*	Wheat bran	0.9	[10]
*S. commune *	Wheat straw	7.7	[10]
*Penicillium occitanis*	Acacia seed	7.9	This work

**Table 3:** Comparison of the mannanase production of *P. occitanis* on acacia seeds and coconut meal.

Fermentation	Maximum mannanase activity (U/mL)	Productivity (U/l/h)
*Acacia seeds powder based medium*		
Batch	41.1 ± 2.0	571.1 ±30.5
Fed-batch	76.1 ± 3.1	649.5 ± 53.6

*Coconut meal based medium *		
Batch	14.1 ± 0.8	175.2 ±8.7
Fed-batch	25.2 ± 1.1	178.5 ± 12.2
